# Self-Esteem, Anxiety, and Depression in Older People in Nursing Homes

**DOI:** 10.3390/healthcare9081035

**Published:** 2021-08-12

**Authors:** Sonja Šare, Marija Ljubičić, Ivana Gusar, Samir Čanović, Suzana Konjevoda

**Affiliations:** 1 Medical School Ante Kuzmanića Zadar, University of Zadar, Franje Tuđmana 24G, 23000 Zadar, Croatia; sonja.sare@gmail.com; 2 Department of Health Studies, University of Zadar, Splitska 1, 23000 Zadar, Croatia; igusar@unizd.hr (I.G.); canovicsamir@yahoo.com (S.Č.); suzana.konjevoda@gmail.com (S.K.); 3 Department of Pediatrics, General Hospital Zadar, Bože Peričića 5, 23000 Zadar, Croatia; 4 Department of Ophthalmology, General Hospital Zadar, Bože Peričića 5, 23000 Zadar, Croatia

**Keywords:** self-esteem, anxiety, depression, older people, nursing home care

## Abstract

Background: Social environment and type of care may influence mental health in late life. The aim of this study was to assess the associations between depression, anxiety, and self-esteem in older people. Methods: The study evaluated mental health in people older than 65 years of age in Zadar County, Croatia. The participants were interviewed using the Patient Health Questionnaire, the Generalized Anxiety Disorder Scale, and the Rosenberg Self-Esteem Scale. Multiple linear regressions were used to assess the correlations between depression, anxiety, and self-esteem. Results: Compared with elderly people living in their own homes, elderly people in nursing homes reported lower self-esteem, higher depression, and higher anxiety. The level of self-esteem decreased with increased levels of depression. Anxiety was significantly associated with depression, while depression strongly increased with an increase in anxiety. Older age was associated with depression, while widowhood was a negative predictor of self-esteem. Institutionalization was borderline significantly associated with anxiety, while gender was associated with anxiety. Conclusions: The study found associations between self-esteem, anxiety, and depression in the elderly. The strategy of humanization in nursing care for older people should be applied equally in nursing homes and in community-dwelling states. Public health programs aimed at maintaining the mental health of older people are needed.

## 1. Introduction

Health, well-being, and prosperity are priorities for aging well [1]. The experience of aging varies per person [2], and physical and mental health have an important influence on the perception of aging and the level of a person’s independence [1,3,4]. Maintaining a positive attitude toward aging, under which aging is not associated with illnesses and exhaustion but rather with new experiences, is of key importance for the health of older people [5]. Nevertheless, not all older people have auspicious life circumstances, a social environment, and good health. In those circumstances, a person’s social context can increase their exposure to stressful stimuli and has negative repercussions on the perception of aging and quality of life [6].

Social environment and type of care may influence an individual’s adaptation to changes that old age brings [4]. However, studies comparing institutional care to home care found noticeably inconsistent results [7]. For example, studies have found that the quality of life in older people living in their own home is higher in comparison with people living in nursing homes (NHs) [8]. Indeed, facing possible institutionalization can result in the loss of self-control in older people, which causes feelings of powerlessness, a lack of motivation, anxiety, and social withdrawal. In addition, leaving one’s own home is one of the most dramatic events in old age [9], which may provoke dissatisfaction, anxiety, and depression, and may negatively impact an individual’s perception of their quality of life. Some studies have confirmed that older people living in an institution have lower self-esteem and worse physical health than people living in their own homes [10]. On the contrary, better social support, higher self-esteem, and a decreased sense of loneliness are more prominent in people living in their own homes [11]. Although living in a home environment may have a positive connotation, whether older people can benefit from living at home depends on many factors [8]. Substandard housing conditions were found to be associated with the risk of losing independence in daily activity performance as well as becoming socially isolated [12]. On the contrary, family support in one’s own home can improve physical and mental health as well as social relationships among older people [13]. On the other hand, even though older people living in NHs enjoy more tangible support from health care professionals and the community [13], there is a lack of systematic understanding of the impact of the environment on the regulation of self-value in older people [14].

Self-esteem, as a regulation of self-value, is important at all stages of life, especially in older people [14]. Self-esteem is an indicator of mental health, mature personality, and a person’s adaptability. Furthermore, low self-esteem is closely related to self-worth, low life satisfaction, loneliness, depression, and anxiety [15]. Depression is a multidimensional concept of social, psychological, and biological factors characterized by cognitive content, sadness, loss of interest or pleasure, tiredness, feelings of guilt or low self-worth, insomnia, loss of appetite, poor concentration, weakening of life energy, low level of physical function, and even cancer and chronic diseases [16,17]. Studies have suggested that anxiety is the most common psychological state among older people living in NHs and that anxiety is associated with depression and cognitive impairment [18,19]. Despite these findings, studies on the association between anxiety, depression, and self-esteem in older people and people living in NHs are scarce.

The aim of this research was to assess the association between depression, anxiety, and self-esteem in older people living in NHs. We hypothesized that the level of anxiety, depression, and self-esteem would be different depending on whether the residence was in a nursing home or in their own home environment. If this assumption is accurate, it is possible that a higher level of anxiety and depression is linked to a lower level of self-esteem in NH residents compared with community-dwelling older people.

## 2. Materials and Methods

### 2.1. Sample and Procedure

This study evaluated mental health in people older than 65 years of age in Zadar County, Croatia, from April to June 2018. The institutionalized respondents were recruited from an NH with 330 respondents. The older people living in their own homes were recruited from the health center in Zadar County, which provides health care to about 36,960 people older than 65 years of age [20]. There were 26,860 older people under the supervision of patronage nurses [20]. During the research period, 9 patronage nurses visited 240 older people in different areas of Zadar County. The exclusion criteria were findings of severe psychiatric disease (such as schizophrenia, depression, dementia, personality disorders, psychotic disorders, obsessive-compulsive, bipolar, and other related disorders) or intellectual disability in the medical records and confirmed by a psychiatrist, people who lost consciousness, and people at the end of their life. Medical records were available for both the NH and community-dwelling older adults. Based on the exclusion criteria and the decline in participation, we excluded 270 respondents (response rate: 52.6%). After the exclusion, two groups of respondents were defined: older people living in an NH (115 women and 35 men; NH group (NH)) and older people living in their own homes (100 women and 50 men; control group (CG)).

Before the beginning of the study, one researcher (S.Š.) instructed the patronage nurses on how to interview the older people. During the home visits, the patronage nurses checked the willingness of the older people to be included in the study. Social workers informed NH respondents about the study and the researchers’ arrival to their rooms. Face-to-face interviews with NH respondents were performed by investigators (the authors), with one researcher and one respondent present per interview. After checking the willingness of participants, all volunteers signed the informed consent forms and were informed of privacy protection. The investigators read multiple choice questions to each respondent individually and recorded the answers obtained from the questionnaires.

The research was approved by the Ethics Committee of the Zadar Nursing Home (02-01/18) and the Zadar County Community Health Center (01-2040/2018). All respondents gave informed consent for inclusion before participating in the study. The research is in compliance with the ethical standards of the Declaration of Helsinki.

### 2.2. Measures

For sociodemographic data analysis, a questionnaire was produced containing information on gender, age, marital status, professional qualifications, living environment housing arrangement and care, as well as the length of residence in the institution. The respondents’ age was qualified as young old (65 to 74 years of age), middle old (75 to 84 years), or oldest old (85 years and older).

The Patient Health Questionnaire (PHQ-9) was used to evaluate depression levels in the last 2 weeks. The scale consists of seven items on the Likert scale from 0 to 3 (0 = not at all, 1= for a couple of days, 2 = more than half of the days, and 3 = almost every day). Different levels of depression are established depending on the final score: minimal depression (0 to 4), mild depression (5 to 9), moderate depression (10 to 14), moderately severe depression (15 to 19), and severe depression (20 to 27) [21].

The Generalized Anxiety Disorder Scale (GAD-7) was used to measure anxiety levels in the last 2 weeks. The scale consists of seven items on the Likert scale from 0 to 3 (0 = not at all, 1 = for a couple of days, 2 = more than half of the days, and 3 = almost every day). Different levels of anxiety are established depending on the final score: minimal anxiety (0 to 4), mild anxiety (5 to 9), moderate anxiety (10 to 14), and severe anxiety (15 to 21) [22].

Self-esteem was tested by the Rosenberg Self-Esteem Scale (RSES), measuring the global value-orientation to oneself, i.e., self-perception of one’s own personality. The scale consists of ten items: five are defined positively, and the remaining five are negatively oriented. Negatively oriented items are scored reversely. The total score is obtained by adding points on a five-point Likert scale (1 = strongly disagree and 5 = strongly agree). The possible range of results varies from 10 to 50 points. A high score reflects high self-esteem [23,24].

### 2.3. Statistical Analysis

The gathered data were processed using SPSS Statistic v21.0 (IBM, Armonk, NY, USA). The reliability assessment was conducted based on the Cronbach alpha coefficient. The Kolmogorov–Smirnov test was used to test the distribution norm. We calculated the median and interquartile range for numeric variables because the data distribution was non-normal. For categorical variables, we used the Chi-square test (number and percentage), while for the single sample with categorical variables (such as environment, place of living, and household for the control group; residence time in NHs and type of accommodation in NHs for the NH group), we used the Chi-square goodness-of-fit test. To examine the differences between the variables, the Chi-square test and Mann–Whitney U test were used. A recoded Pearson correlation coefficient (non-parametric partial correlation) was used to adjust for age and to analyze the correlation between the variables.

The associations between self-esteem, anxiety, and depression were made by creating linear regression models. The independent variable in each regression model was the NH study group (the control group was the referent group), gender (females were the referent group), education (educated was the reference group), widowhood (married was the reference group), never married (married was the reference group). Values of *p* < 0.05 were considered statistically significant values.

## 3. Results

### 3.1. Sociodemographic Data

The sociodemographic characteristics are shown in Table 1. The average age of all the respondents was 80 years (Me = 80.5; IQR = 12). Women dominated in both groups. The institutionalized respondents were somewhat older than the control group, mainly belonging to the middle-old and oldest-old age groups, and lower educational level (*p* < 0.001 for all). In the NH group, widows/widowers (*p* < 0.001) who had been in the NH less than 5 years, mostly in double rooms (*p* < 0.001), dominated. Older people in the CG mostly lived in their own homes with family members (*p* < 0.001), with an equal distribution of rural and urban environments (*p* = 0.021) (Table 1).

### 3.2. The Level of Depression, Anxiety, and Self-Esteem in Older People

Compared with older people living in their own homes, people living in NHs reported lower self-esteem (*p* = 0.002), higher depression (*p* < 0.001), and higher anxiety (*p* < 0.001) (Table 2). No differences between genders were found in the level of self-esteem. Nevertheless, both men and women in the NH group had lower self-esteem in comparison to those in the control group (Table 2). Men and women in the NH group showed higher levels of depression than those in the control group, and similar trends were found for anxiety levels (*p* < 0.001). However, women in the control group showed higher levels of anxiety (*p* = 0.002) and depression (*p* = 0.049) than men. This trend was not found in the NH group (Table 2).

Mild depression and moderate anxiety prevailed in the NH group, whereas both depression and anxiety levels were low in the control group (Table 3). The internal reliability of all of the questionnaires was good (α = 0.82 for PHQ-9, α = 0.84 for GAD-7, and α = 0.82 for RSES).

### 3.3. The Association between Self-Esteem, Anxiety, and Depression

A significant positive correlation was found between anxiety and depression (R = 0.75; *p* < 0.001). A negative moderate correlation was found between self-esteem and anxiety (R = −0.50; *p* < 0.001) and between self-esteem and depression (R = −0.63; *p* < 0.001; Table 4).

The linear regression confirmed that the level of self-esteem decreased with an increase in the level of depression (ß = −0.59; *p* < 0.001). Similarly, the level of depression increased with a decrease in the level of self-esteem (ß = −0.35; *p* < 0.001). Age, gender, and institutionalization were not associated with changes in self-esteem. However, widowhood was the only sociodemographic variable negatively associated with self-esteem and confirmed as a negative predictor (ß = −0.12; *p* = 0.048; Table 5).

Institutionalization showed a borderline significant negative association with anxiety (ß = −0.10; *p* = 0.055), while male gender was positively associated with anxiety (ß = 0.10; *p* = 0.013). Moreover, depression strongly increased with the increase in anxiety (ß = 0.69; *p* < 0.001), while anxiety was positively associated with depression (ß = 0.55; *p* < 0.001). Older age was significantly associated with depression (ß = 0.13; *p* = 0.003), whereas other sociodemographic factors such as gender, level of education, marital status, and living in an NH were not significantly related to the increase in depression (Table 5).

## 4. Discussion

Our results confirm the associations between self-esteem, anxiety, and depression in older people. Self-esteem was negatively associated with depression, and depression was a moderate predictor for lower self-esteem. A higher level of depression in older people was associated with age, lower self-esteem, and higher anxiety. After controlling for differences between NH and community-dwelling older adults, the regression analyses demonstrated that living in an NH was not associated with self-esteem or depressive symptoms. A somewhat unexpected result was the borderline significant association between anxiety and living in an NH, which indicated lower levels of anxiety in the NH group. This may indicate that in comparison to the community-dwelling older adults, the NH residents have an increased risk of poor mental health. This suggests that some independent factors could be related to anxiety, depression, and self-esteem. For example, older people who move into NHs often have less social support than older adults who are able to live in their own homes with family. Older people who move into NHs are generally unhealthier, suffer from dementia, and have a higher degree of functional impairment [7,25,26,27]. Both physical and mental health conditions can influence psychological wellness, disrupt self-esteem, and lead to the development of anxiety disorders and depression in older people [28]. Our results show a statistically significant association between lower self-esteem and widowhood. This correlation suggests that the loss of a spouse is a possible predictive factor of lower self-esteem and, thus, of increased physical and social isolation [29]. We found that moderate anxiety and mild depression prevail among older people in NHs. These results suggest that this type of care can have a negative influence on the psychological state of a person and that it can represent a high-risk factor for depression [30]. Although our results indicate a higher presence of negative indicators of mental health in respondents living in NHs, they do not confirm a significant correlation between the social and demographic factors and the level of self-esteem and depression. Since the cross-sectional study and regression models did not confirm the influence of institutionalization in the variables, such presumptions need further research.

The association between depressive symptoms, gender, age, psychological distress, and social isolation are known [31]. The lack of correlation between self-esteem and socio-demographic factors such as age, gender, social environment, and marital status in our study suggests that self-esteem is a construct that is relatively stable and preserved in a specific personality [32]. The NH residents were older than people in a home environment and showed higher levels of depression and lower self-esteem. Indeed, Wagner et al. found that the process of self-esteem is relatively stable until people reach their late 80s and early 90s, when self-esteem starts to decline [33]. Orth et al. also found that self-esteem peaks at age 60 and remains constant until age 70; then, it slightly declines until age 90 and strongly declines until age 94 [32]. The association between widowhood and self-esteem in our study suggests that the loss of a spouse and the loss of relationships with the deceased’s associates may provoke social isolation and have negative implications on family interaction and self-esteem in older people [29]. Indeed, studies have shown that widowed men are more depressed than widowed women [34] and that women adapt quicker to single life [35]. In our research, the respondents in the NH group were of older age, had lower educational levels, and were mostly widowed. However, those factors did not significantly influence the levels of self-esteem, anxiety, and depression. It can be assumed that living in an NH without a spouse can cause loneliness, can have a negative influence on self-esteem, and can incite the development of depressive symptoms. A regression model confirmed that male gender was positively associated with anxiety, which may potentially predict cognitive decline and lower functioning in older men [18]. However, gender differences in self-esteem were not found between any of the groups of older people. Women living in a home environment were slightly more anxious and depressed than men. On the other hand, these women had lower anxiety and depression and higher self-esteem than women in NHs. It is possible that women living in a home environment are more anxious because they show a higher degree of emotional inclusion and interpersonal sensitivity when facing problems and everyday duties [36]. Daily obligations and loneliness may reinforce concern, especially if a woman is widowed [37]. In addition, these women take care of the household, their own health and sometimes the health of their partner, undergo regular medical examinations, and may potentially take medications at the proper dose and intervals during the day. While this can result in a higher sense of self-efficacy and autonomy, it can still be stressful and foster concern for the future [36]. In NHs, the medical staff takes care of many of these obligations. It is possible that prescription drugs, which can help to reduce mood swings in older people, are slightly more common in NHs than in the home environment [38], but these data from the medical record of our respondents did not indicate this possibility. Additionally, communication with coevals and interactions between residents and nursing staff in the NH affects and improves the adaptation and socialization of older people in NHs, as confirmed by the literature [29,39]. These points can be associated with non-significant lower anxiety levels in the NH group.

Previous studies on self-esteem changes in adults have shown inconsistent results, linking self-esteem to the self-perception of older people. Some authors have concluded that self-esteem is not connected to chronological age but rather to social integration and adaptability to life events, including physical and cognitive decline. According to these authors, the aging process does not necessarily result in a decrease in self-esteem, regardless of the decline in numerous areas of mental functioning and other activities. Moreover, according to some studies, a higher level of education also contributes to higher self-esteem [10], a better understanding of all changes that occur in the aging process, and adaptation [40]. In a Brazilian study conducted on older people, the level of self-esteem was defined by the quality of life, termination of professional activities, retirement, and health status [41]. Indeed, lower self-esteem in Croatian older people living in NHs was also found in a study comparing self-esteem and quality of life between NH residents and older people living at home [42].

Although self-esteem and depression are closely connected, there is still no consistent evidence to prove this claim [43]. Different models found in the literature link and explain the connection between self-esteem, anxiety, and depression. In the vulnerability model, low self-esteem is considered a risk factor for depression and anxiety, while the scar model suggests that low self-esteem is an outcome of depression rather than its cause [44]. However, it is still not sufficiently known whether the mentioned models, which are specific to depression, can be applied to anxiety as well. An analysis of a longitudinal study on the correlation between self-esteem, anxiety, and depression confirmed the vulnerability model [45]. Therefore, the effect of self-esteem on depression has been proven to be considerably stronger than the effect of depression on self-esteem [46]. Our results showed the effect of depression on self-esteem and vice versa, highlighting the unbreakable link between them. Unlike depression, the effect of anxiety on self-esteem is not significant. However, the mentioned variables have not been equally represented in research, and the correlation between self-esteem and anxiety has been scarcely discussed in the literature [44].

According to our knowledge, studies on the correlation between the aforementioned variables are not significantly represented in older populations. Furthermore, the research shows that generalized anxiety disorders are more frequent than other disorders, but there is surprisingly little research in this area. Due to the lack of research, one can conclude that anxiety is an unrecognized disorder that seriously affects quality of life [47]. As for depression, studies have confirmed the prevalence of depression symptoms in around half of the older population, predominantly females living in NHs, indicating that a lack of social support and institutionalization are connected to depressive symptoms [48,49,50]. Furthermore, risk factors for DEPRESSION are female gender and older age [51]. Unlike gender, age was found to be a predictor of depression in our research. As mentioned before, older age had a considerable influence on the incidence of a higher level of depression due to low self-esteem and higher levels of anxiety. Likewise, the level of self-esteem decreased with an increase in the level of depression. In research on the correlations between self-esteem, anxiety, and depression, it is important to emphasize that anxiety is an important affective variable, and there is a clinical connection between general health, anxiety, and depression [52,53,54]. On the contrary, a small number of longitudinal studies have specifically targeted the correlation between self-esteem and anxiety [45,55].

It is also necessary to point out that older people with more psychosocial stressors and those who need stronger emotional support and have low awareness of their own abilities have a higher risk of anxiety [56]. Respondents living in NHs, even those in good health, are more prone to feelings of anxiety and depression [56], insomnia, loss of appetite, and bad mood [31]. Accordingly, it is necessary to plan interventions that will increase the self-esteem of older people [45,54,57,58]. In addition, social interactions can reduce the risk of anxiety and the development of depression [59]. Communication with other people, participating in group activities, remembering positive past events, and enhancing positive feelings are just some of the social interventions targeting better social relations, and friendships may improve the mental health and quality of life in older people regardless of their place of residence [60]. For older people in nursing homes, family connection in the form of frequent visits by direct contact or videoconferencing technology should be encouraged [13,60,61].

According to our knowledge, this is one of the only studies analyzing self-esteem, anxiety, and depression in older people in NHs compared with older people living at home. However, this study has certain limitations that make it difficult to generalize the results. First, this is a cross-sectional study, and it is neither possible to prove the causality of lower self-esteem in institutionalized respondents nor possible to prove causes of higher anxiety and depression. Second, it is possible that the relatively small sample size affected the statistical power and resulted in the absence of statistical significance in the regression models. Third, respondents were not chosen randomly. Instead, convenience sampling was used. Fourth, only one NH participated in this study. This institution is the largest public NH in Zadar County, while other existing institutions are private and take care of a very small number of older people or people with psychiatric disorders. Fifth, we did not test the health and psychological state of the respondents at the moment of their arrival to the institution, their personality traits, their level of social support, or their socioeconomic status, which can be predisposing factors to mental health problems such as anxiety and depression [62,63]. Sixth, medical records often do not precisely document anxiety and depression accurately (many cases are missed). This absence does not mean that anxiety and depression identified by screening scales during a later interview are new. Accordingly, longitudinal testing is needed to assess the influence of psychosocial factors and institutionalization on the incidence of depression, anxiety, and low self-esteem.

Despite the mentioned limitations, this study furthers the understanding of older people’s mental health, confirming the correlation of higher levels of depression; higher levels of anxiety and lower self-esteem with old age; as well as the possible connection of anxiety, depression, and self-esteem with institutionalization. However, longer-term studies are needed to clarify the influence of environment on the mental health and self-esteem of older people, especially those living in NHs. Some strategy programs such as promoting positive well-being may assist in the assessment, early identification, and monitoring older people with symptoms of disrupted mental health [64]. It is imperative that preventive strategies be developed to preserve mental health, to prevent depression, and to maintain high self-esteem in older people.

## 5. Conclusions

This study confirms the associations between self-esteem, anxiety, and depression in the elderly. Our data suggest that low self-esteem is associated with a higher level of depression, which increases with age. Male gender and living in an NH can be a predictor for anxiety, while widowhood may also affect self-esteem. This can have negative implications on the mental health of older people, especially those living in institutions. For this reason, it is necessary to recognize the symptoms of depression in older people as soon as possible. The strategy of humanization in nursing care for older people should be applied equally in NHs and community-dwelling states. This would reduce the incidence of mental disorders and positively impact the aging process. Public health programs aimed at maintaining mental health in elderly people are needed.

## Figures and Tables

**Table 1:** Sociodemographic characteristics of older people.

Variable	Control Group (*n* = 150)	Nursing Home Group (*n* = 150)	*p*
Gender; *n* (%)			
male	50 (33.3)	35 (23.3)	0.055 *
female	100 (66.7)	115 (76.7)	
Age; *n* (%)			
young old (65–74 years)	71 (47.3)	7 (4.7)	
middle old (75–84 years)	60 (40.0)	65 (43.3)	<0.001 *
oldest old (>85 years)	19 (12.7)	78 (52.0)	
Marriage status; *n* (%)			
in marriage	82 (54.7)	8 (5.3)	<0.001 *
never married	12 (8.0)	12 (8.0)	
widow/widower	56 (37.3)	130 (86.7)	
Education; *n* (%)			
without education	21(14.0)	69 (46.0)	
elementary school	77 (51.3)	34 (22.7)	<0.001 *
high school	39 (26.0)	32 (21.3)	
university education	13 (8.7)	15 (10.0)	
Residence time in nursing home; *n* (%)			
≤5 years		114 (76.0)	<0.001 ^†^
6–10 years		31 (20.7)	
≥11 years		5 (3.4)	
Type of accommodation in NH; *n* (%)			
one bed room		57 (38.0)	
room with two beds		81 (54.0)	<0.001 ^†^
room with three beds		12 (8.0)	
Environment; *n* (%)			
village	58 (38.6)		
city	58 (38.6)		0.021 ^†^
island	34 (22.6)		
Place of living; *n* (%)			
own house	123 (82.0)		<0.001 ^†^
building	27 (18.0)		
Household; *n* (%)			
living with family	120 (80.0)		<0.001 ^†^
live alone	30 (20.0)		

Note: NH = nursing home; Me = median; IQR = interquartile range; * Chi-square; ^†^ Chi-square goodness-of-fit test.

**Table 2:** Measures of self-esteem, anxiety, and depression in older people.

Variable	Control Group (*n* = 150)		Nursing Home Group (*n* = 150)		*p* *	*p* ^†^
	Me(IQR)	Mean Rank	Me(IQR)	Mean Rank		
Self-esteem (α = 0.82)	40.0 (12.3)	165.68	37.0 (9.0)	135.32	0.002	
male	40.0 (12.5)	48.37	35.0 (7.0)	35.33	0.016	0.353
female	40.0 (12.8)	117.22	37.0 (9.0)	99.98	0.043	
Anxiety (α = 0.84)	6.0 (8.0)	130.97	10.0 (8.3)	170.03	<0.001	
male	4.0 (7.5) *	37.93	9.0 (11.0)	50.24	0.023	0.001
female	8.0 (9.0) *	96.20	10.0 (8.0)	118.27	0.009	
Depression (α = 0.82)	5.0 (8.0)	132.28	8.0 (8.0)	168.72	<0.001	
male	3.0 (6.3)	37.61	9.0 (9.0)	50.70	0.016	0.003
female	6.0 (8.0)	96.51	8.0 (8.0)	117.99	0.011	

Note: Me = median; IQR = interquartile range; α = internal reliability; *p* * = Mann–Whitney U test between control group and nursing home group; *p* ^†^-Mann–Whitney U test by gender.

**Table 3:** Distribution of anxiety and depression in older people, *n* (%).

Variable	Control Group (*n* = 150)	Nursing Home Group (*n* = 150)	*p* *
Anxiety			
minimal	65 (43.3)	29 (19.3)	<0.001
mild	29 (19.3)	43 (28.7)	
moderate	39 (26.0)	50 (33.3)	
severe	17 (11.3)	28 (18.7)	
Depression			
minimal	65 (43.3)	36 (24.0)	0.002
mild	47 (31.3)	51 (34.0)	
moderate	21 (14.0)	35 (23.3)	
moderately severe	15 (10.0)	19 (12.7)	
severe	2 (1.3)	9 (6.0)	

Note: * Chi-square test.

**Table 4:** Correlations between self-esteem, anxiety, and depression for all participants (*n* = 300).

		Anxiety	Depression
Self-esteem	R *	−0.500	−0.627
	*p*	<0.001	<0.001
Anxiety	R *		0.752
	*p*		<0.001

* Recoded Pearson correlation coefficients (non-parametric partial correlation to adjust for age).

**Table 5:** Regression analysis for variables predicting self-esteem, anxiety, and depression.

	Self-Esteem		Anxiety		Depression	
	ß	*p*	ß	*p*	ß	*p*
Nursing home group (controls are referent group)	−0.27	0.753	−1.00	0.055	0.57	0.269
Age	0.03	0.559	−0.04	0.235	0.09	0.003
Gender (male) (female are referent group)	0.45	0.559	1.16	0.013	−0.33	0.470
Without education (education are referent group)	−0.60	0.405	0.31	0.599	−0.16	0.775
Widowhood (in marriage are referent group)	−1.91	0.048	1.16	0.137	0.37	0.626
Never married (married are referent group)	0.37	0.776	−0.55	0.205	0.41	0.334
Self-esteem	N/A		−1.00	0.242	−0.26	<0.001
Anxiety	−0.11	0.242	N/A		0.60	<0.001
Depression	−0.77	<0.001	0.63	<0.001	N/A	
R	0.67		0.77		0.82	
R^2^	0.44		0.59		0.67	
F-statistic	F(8, 291) = 29.93, *p* < 0.001		F(8, 291) = 52.53, *p* < 0.001		F(8, 291) = 74.77, *p* < 0.001	

Note: ß = standardized coefficient; *p* = *p*-value; R = multiple correlation coefficient; R^2^ = coefficient of determination, total variation in the dependent variable explained by the independent variables; F = statistically relationship to dependent variable; N/A = not available.

## Data Availability

The data presented in this study are available from the corresponding author upon request.
